# Non-invasive Spatial Mapping of Frequencies in Atrial Fibrillation: Correlation With Contact Mapping

**DOI:** 10.3389/fphys.2020.611266

**Published:** 2021-01-06

**Authors:** Miguel Rodrigo, Kian Waddell, Sarah Magee, Albert J. Rogers, Mahmood Alhusseini, Ismael Hernandez-Romero, Alejandro Costoya-Sánchez, Alejandro Liberos, Sanjiv M. Narayan

**Affiliations:** ^1^Stanford University School of Medicine, Stanford, CA, United States; ^2^ITACA Institute, Universitat Politècnica de València, Valencia, Spain; ^3^Department of Signal Theory and Communications, Rey Juan Carlos University, Móstoles, Spain

**Keywords:** atrial fibrillation, driver, dominant frequency, non-invasive mapping, Electrocardiographic imaging, basket mapping

## Abstract

**Introduction:** Regional differences in activation rates may contribute to the electrical substrates that maintain atrial fibrillation (AF), and estimating them non-invasively may help guide ablation or select anti-arrhythmic medications. We tested whether non-invasive assessment of regional AF rate accurately represents intracardiac recordings.

**Methods**: In 47 patients with AF (27 persistent, age 63 ± 13 years) we performed 57-lead non-invasive Electrocardiographic Imaging (ECGI) in AF, simultaneously with 64-pole intracardiac signals of both atria. ECGI was reconstructed by Tikhonov regularization. We constructed personalized 3D AF rate distribution maps by Dominant Frequency (DF) analysis from intracardiac and non-invasive recordings.

**Results:** Raw intracardiac and non-invasive DF differed substantially, by 0.54 Hz [0.13 – 1.37] across bi-atrial regions (*R*^2^ = 0.11). Filtering by high spectral organization reduced this difference to 0.10 Hz (cycle length difference of 1 – 11 ms) [0.03 – 0.42] for patient-level comparisons (*R*^2^ = 0.62), and 0.19 Hz [0.03 – 0.59] and 0.20 Hz [0.04 – 0.61] for median and highest DF, respectively. Non-invasive and highest DF predicted acute ablation success (*p* = 0.04).

**Conclusion:** Non-invasive estimation of atrial activation rates is feasible and, when filtered by high spectral organization, provide a moderate estimate of intracardiac recording rates in AF. Non-invasive technology could be an effective tool to identify patients who may respond to AF ablation for personalized therapy.

## Introduction

Pharmacological and surgical therapies for atrial fibrillation (AF) continue to have suboptimal outcomes despite advances in anatomical mapping and catheter technology. Isolation of the pulmonary veins (PVI) is the cornerstone of ablation, yet its 1 year success is 40–70% depending on population, and it is unclear how this can be improved. The identification and elimination of regions of rapid atrial activity are plausible mechanistic sites and indicate drivers in optical imaging of human AF (Hansen et al., 2015) and clinical studies (Miller et al., 2017; Honarbakhsh et al., 2019). These fastest activated regions may lie near scar or fibrosis (Swartz et al., 2009), and can be represented by Dominant Frequency (DF) (Atienza et al., 2014), which identifies the activation rate as the greatest spectral contribution. It would be useful to identify such atrial regions non-invasively to characterize AF patients, and potentially to plan whether ablation should include PVI alone, more extensive ablation, or potentially other therapeutic strategies.

Electrocardiographic Imaging (ECGI) enables non-invasive reconstruction of epicardial electrical activity and may non-invasively characterize AF. ECGI has been used to identify regions in AF with reentrant and focal patterns to guide ablation (Haissaguerre et al., 2014), and shows regional differences in rate and sites of high dominant frequency (DF) (Pedrón-Torrecilla et al., 2016). Nevertheless, the accuracy of ECGI has been questioned (Cluitmans et al., 2017; Duchateau et al., 2018), and the DF of AF estimated from ECGI has never been calibrated against direct measurements from contact catheters. Finally, existing commercial ECGI systems use 252 leads which introduce practical limitations for bedside use, and increase the chance that electrodes may lose contact at some regions of the torso.

We set out to calibrate ECGI obtained using our published practical reduced-lead method, against simultaneously recorded panoramic intracardiac measurements in AF. We also correlated analyses to the clinical endpoint of acute ablation success resulting in termination of AF.

## Materials and Methods

### Patient Inclusion

We recruited 47 consecutive patients undergoing AF ablation with simultaneous intracardiac basket and ECGI torso mapping at two centers: Stanford Hospital (SH, CA, United States, *N* = 17) and Hospital General Universitario Gregorio Marañón (HGUGM, Madrid, Spain, *N* = 30). Patients from Stanford University (*N* = 17) received PVI and also substrate guided ablation. In this approach, lesions were applied for 15 to 30 s at sites of focal or rotational activity in AF (Miller et al., 2017) identified by a commercial mapping system (RhythmView, Abbott) from basket recordings. Patients from Spain received only PVI, regardless of perceived substrate. Both protocols were approved by each local Institutional Ethics Committee and all patients gave informed consent.

### Electrophysiological Study

Classes I and III antiarrhythmic medications were discontinued for > 5 half-lives prior to study (> 30 days for amiodarone). Catheters were advanced to the right atrium (RA), coronary sinus, and transseptally to left atrium (LA). In patients arriving in sinus rhythm, AF was induced using burst pacing. Contact basket catheters (64 poles) were positioned in RA, then LA for AF mapping, based on 3-dimensional electroanatomic imaging (NavX, St Jude Medical, Sylmar, CA, United States).

### Data Acquisition

**FIGURE 1:**
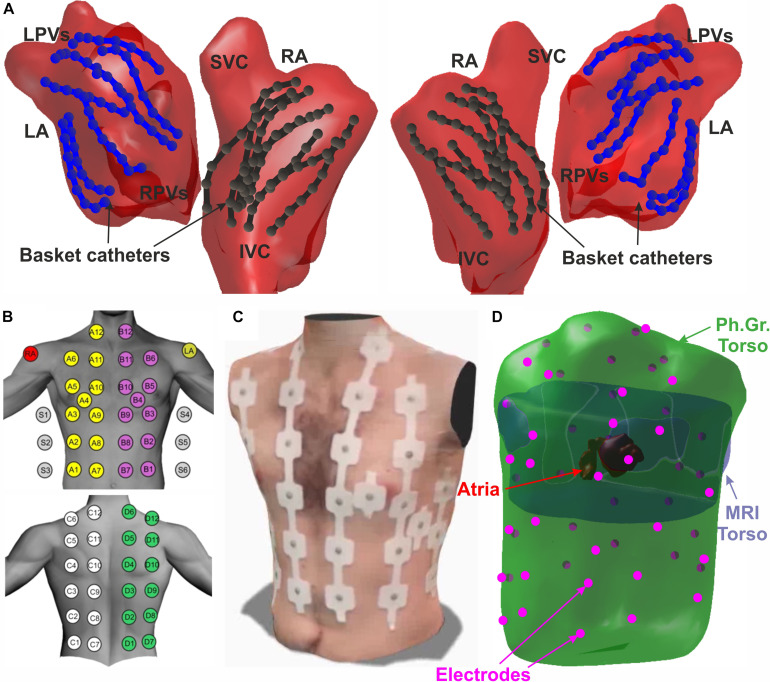
Schematic view of the experiment set-up. **(A)** Atrial anatomy (red) with 2 basket catheters in left (blue) and right (black) atria. **(B)** Atrial anatomy segmentation from MRI scan. **(C)** Surface 57-ECG electrode distribution. **(D)** 3D meshes of the MRI torso (purple), photogrammetry torso (green), atrial anatomy (red), and BSPM electrodes (pink).

Unipolar electrograms (EGM) from basket catheters were recorded at 1 kHz sampling frequency and filtered at 0.05 to 500 Hz. Raw electrograms comprising 64 basket and other intracardiac channels (e.g., coronary sinus) and 12-lead ECG were exported from Bard (LabSystem Pro), Prucka (GE Cardiolab) or Boston Scientific (Clearsign^TM^) recorders for off-line analysis. Basket electrode positions and atrial anatomy meshes were extracted from the electro-anatomical navigation system (Ensite NavX System) that enabled atrial anatomy reconstruction (Figure 1A).

MRI/CT images were acquired 2–3 days prior to the ablation procedure, and segmented to provide atria and torso anatomy using ITK-SNAP (Yushkevich et al., 2006; Figure 1B). Surface ECG positions were registered on torso anatomy as we have described (Rodrigo et al., 2020). Anatomical models obtained with the different technologies were co-registered by using an algorithm based on rigid transformations guided by fiducial points manually marked in both atrial models (PVs, LAA, RAA, superior vena cava (SVC) and inferior vena cava (IVC)) or torso models (anterior and posterior axillae, nipples, low scapula and xiphoid appendix) (Rodrigo et al., 2020).

Electrocardiographic imaging was recorded with surface ECG leads at electrophysiological study. ECG electrodes were distributed as follows: 24 electrodes on the anterior, 24 on the posterior, 3 on each lateral side of the torso and 3 extra leads to obtain Wilson Central Terminal (Figures 1C,D). We used the same filtering as for intracardiac electrograms (0.05 to 500 Hz band-pass at 1 kHz) as previously described (Rodrigo et al., 2020).

### Data Analysis

Simultaneous basket EGM and surface ECG signals were analyzed from 160 AF episodes (3 [2 – 5] per patient). Signals with duration 4.9 ± 1.4 s were used for Dominant Frequency (DF) analysis. A total of *N* = 4566 pairs of Intracardiac and non-invasive signals were analyzed. Surface electrode recordings were discarded if noisy or poor contact defined as those in which QRS complexes had signal-to-noise ratio < 0 dBs.

For intracardiac analysis, we calculated bipolar electrograms by subtracting unipolar electrograms for successive pairs of electrodes along basket splines (for instance A1-2, A3-4). Intracardiac DFs were then calculated from the Power Spectral Density (PSD) curves of these bipolar EGM signals using a convolutional filter composed by a band-pass filter (2 to 20 Hz, Butterworth) (Rodrigo et al., 2014) and a Botteron and Smith filter (Botteron and Smith, 1996). The PSD of both filtered signals was obtained by Welch Periodogram (50% overlapping, 2 s-length Hamming window, 65.536 points) and both PSDs were multiplied to get the final convolutional PSD (de la Torre Costa et al., 2016). The dominant frequency was defined as the maximal contribution in the 3 to 8 Hz band, discarding peaks whose sub-harmonic contribution (at DF/2) was higher than 50% of the DF peak amplitude. Spectral organization of EGM signals was calculated using the regularity index (RI), which is the cumulative sum of PSD in a 0.5 Hz bandwidth surrounding the DF peak (± 0.25 Hz) (Skanes et al., 1998; Li et al., 2017).

For ECGI analysis, a QRST removal algorithm based on principal component analysis (PCA) was first applied to each ECG channel (Guillem et al., 2013). Then, the ECG baseline was estimated by decimation of raw signal (sample frequency of 50 Hz) and a posterior low-pass filtering (Butterworth 10th-order, cut-off frequency of 2 Hz). This baseline signal was interpolated to the original sample frequency and then subtracted from the original signal. ECG signals were then low-pass filtered with a 10th-order Butterworth filter with a cut-off frequency of 20 Hz. We estimated the inverse-computed Electrogram signals (ic-EGM) by applying the zero-order Tikhonov’s method on the filtered surface signals over the torso and atrial anatomy. The optimal regularization parameter was chosen at the first local maximum value of the curvature of the L-curve (Rodrigo et al., 2017a). Non-invasive DFs in ic-EGM signals were identified on the PSD of raw reconstructed signals, which were already filtered on the ECG. The PSD of non-invasive signals was obtained by Welch Periodogram (50% overlapping, 2 s-length Hamming window, 65.536 points) and DF and RI was calculated as described for intracardiac recordings.

Median DF value was used to describe the overall DF values for each patient and/or episode. Highest DF (HDF), calculated as the 95% percentile of the intracardiac or non-invasive DF measures, respectively, were used to describe the fastest activation rate avoiding possible harmonic detection.

### Ablation

Radiofrequency energy was delivered via an irrigated catheter (Cool-Flex/TactiCath/Sapphire-Blue, St Jude Medical) at 25 to 35 W. All patients received standard-of-care pulmonary vein isolation (PVI). Patients from Stanford center (*N* = 17) also received guided ablation, in whom lesions were applied for 15 to 30 s at sites of focal or rotational activity during AF guided by basket mapping and commercially available software (RhythmView, Abbott). Such potential driver sites were targeted if they were present for > 50% of recorded tracings. Ablation covered areas of 2 to 3 cm^2^ as described by Miller et al. (2017). PVI was performed by circumferential point-by-point ablation or cryoablation of left and right PV pairs (Artic Front, Medtronic Inc.) with verification of PV isolation using dedicated circular mapping catheters.

### Performance Metrics and Statistical Analysis

Different measures were included to compare non-invasive vs. intracardiac DF measures. Relative Difference Measurement Star (RDMS) and Relative Absolute Error (RAE) metrics were used to evaluate the relative difference between intracardiac and non-invasive DF measurements (Figuera et al., 2016). To evaluate the accuracy of non-invasive measures to identify sites of Highest DF (HDF) identified by intracardiac measures, we used the Weighted Underestimation Indicator (WUI), defined as the percentage of the intracardiac HDF region not detected non-invasively as HDF, and the Weighted Over-estimation Indicator (WOI), defined as the falsely detected non-invasive HDF areas as a percentage of the total non-invasive HDF region (Figuera et al., 2016). HDF regions for each AF epoch were defined as regions with DF within 0.5 Hz of HDF (i.e., > HDF −0.5 Hz), calculated for both invasive and non-invasive maps.

Continuous data are represented as mean ± SD, when normally distributed, or median [lower quartile – higher quartile] otherwise. Normality was evaluated using the Kolmogorov–Smirnov test. Comparisons between 2 groups were made with Student *t*-tests for independent samples if normally distributed, or if not normally distributed, with the Mann–Whitney *U*-test. Nominal values were expressed as n (%) and compared with χ^2^ tests. Paired *t*-test was used for paired comparisons with continuous variables. A probability of < 0.05 was considered statistically significant. Repeated-measures one-way ANOVA test was used to compare patient-specific and global linear fits.

## Results

Patient demographics are shown in Table 1. Patients who underwent PVI and driver ablation had more comorbidities than other patients in the proportion of persistent AF cases, duration of AF history and demographics, with a higher rate of AF termination by ablation.

**TABLE 1:** Cohort demographics.

	**All patients**	**PVI + Driver ablation (Stanford)**	**PVI Only (Spain)**	***P*-value (Guided vs PVI)**
**N**	47	17	30	–
**Paroxysmal AF (%)**	20 (43%)	3 (18%)	17 (57%)	**0.009**
**Male (%)**	21 (45%)	14 (82%)	7 (23%)	**<0.001**
**Age (years)**	63 ± 13	67 ± 9	61 ± 14	0.13
**AF history (months)**	55 ± 56	85 ± 70	36 ± 32	**0.01**
**Previous ablations**	1.1 ± 0.9	0.6 ± 1.0	1.4 ± 0.8	**0.004**
**Acute termination (%)**	12 (26%)	7 (41%)	5 (17%)	0.06
**CHA2DS2-VASc Score**	2.4 ± 1.5	2.5 ± 1.5	2.4 ± 1.6	0.80
**Weight (kg)**	78 ± 17	93 ± 14	70 ± 13	**<0.001**
**Risk factors**				
HTA (%)	24 (51%)	12 (71%)	12 (40%)	**0.04**
Diabetes (%)	8 (17%)	5 (29%)	3 (10%)	0.09
Obesity (%)	8 (17%)	4 (24%)	4 (13%)	0.37
Alcohol/Tabaquism (%)	9 (19%)	1 (6%)	8 (27%)	0.08
**Cardiovascular diseases**				
Valvulopaty (%)	13 (28%)	0 (0%)	13 (43%)	**0.001**
CAD (%)	6 (13%)	3 (18%)	3 (10%)	0.45
CHF (%)	5 (11%)	2 (12%)	3 (10%)	0.85
Dilated cardiomyopathy (%)	9 (19%)	6 (35%)	3 (10%)	0.04
**Medications**				
Class I/III antiarrhythmics	21 (45%)	7 (41%)	14 (47%)	0.72
Class II/IV antiarrhythmics	39 (83%)	15 (88%)	24 (80%)	0.47
Angiotensin Converting Enzyme Inhibitors (ACEI)/ Angiotensin Receptor Blockers (ARBs)	14 (30%)	7 (41%)	7 (23%)	0.82

*Bold numbers are the statistically significant differences.*

### Non-invasive Identification of Atrial Activation Rate

**FIGURE 2:**
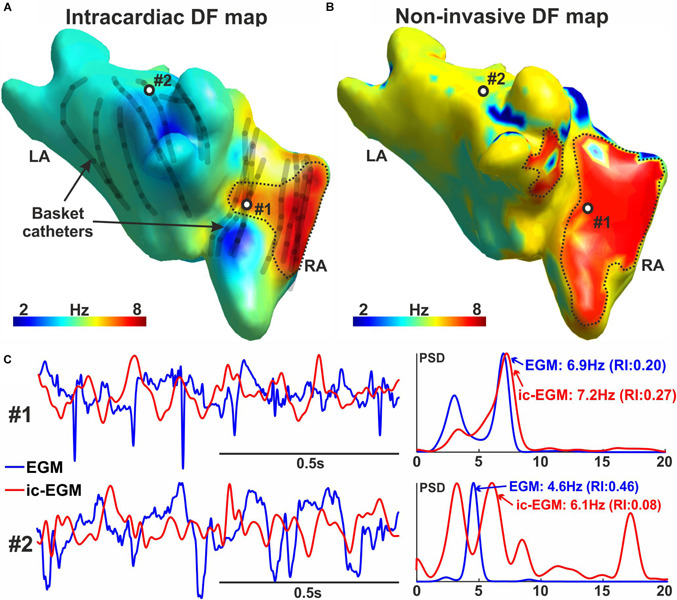
Intracardiac and non-invasive DF map. **(A)** Intracardiac DF map. **(B)** Non-invasive DF map. **(C)** Intracardiac and non-invasive signals and Power Spectral Density (PSD) from points #1 and #2 marked in panels **(A,B)**.

An illustrative example of simultaneous intracardiac and non-invasive DF maps during an AF episode is depicted in Figure 2. In panel A, the DF values obtained from each basket electrode are projected onto the atrial surface and color-coded, with warm colors for higher frequencies. In this unusual episode with 2 basket catheters simultaneously placed in the left and right atria, the RA showed higher DF values of ∼7 Hz in the RA lateral wall, whereas the rest of the RA showed lower DF values ∼6 Hz and the LA ∼4.5–5 Hz.

The non-invasive ECGI AF rate map simultaneous to the basket recording (Figure 2B) shows a similarly located fastest region (7 Hz). This region covers a larger region on ECGI than on intracardiac maps, extending to much of RA and the septal LA. Remaining areas had lower frequencies around 5–6 Hz. Examples of intracardiac and non-invasive traces processed to obtain DF measures are shown in Panel C for signals #1 and #2 marked in Panels A and B, respectively. This patient received PVI only ablation, which did not terminate AF, and this right atrial site was not targeted.

A higher regularity index (RI) indicates that the frequency peak under consideration makes a greater contribution to the selected frequency range. In this study, RI identified intracardiac and non-invasive signals with more reliable DF measurements. In Figure 2C: the signal at position #1 on intracardiac and non-invasive traces showed periodic deflections every 140–150 ms, indicating 6.9 and 7.1 Hz yet with moderate RI = 0.20 and 0.17, respectively. The intracardiac signal at position #2 showed a clear spectral frequency of 4.6 Hz with a high RI value of 0.46, corresponding to observed deflections with period = 220 ms. Conversely, the ic-EGM trace at position #2 showed 2 similar spectral peaks at 3.3 and 6.1 Hz with low RI values of 0.07 and 0.08, respectively, which therefore may not faithfully represent the original signal periodicity. This is observed in the raw ic-EGM signal, in which no clear trend is observed at period = 300 ms (3.3 Hz) nor period = 165 ms (6.1 Hz).

### Systematic Comparison of Raw Non-invasive and Basket DF

Supplementary Figure 1 summarizes the ability of non-invasive DF to estimate intracardiac DF in AF, examined by comparing 4566 non-invasive EGM vs. intracardiac-EGM pairs measured at the closest nodes of the atrial mesh to each intracardiac electrode (overall projection distance 4.4 ± 2.3 mm). Points with better agreement between intracardiac and non-invasive DFs (main diagonal) had higher values for intracardiac and non-invasive spectral regularity index (RI) (Skanes et al., 1998). Overall, the difference between non-invasive and invasive DF was 0.54 Hz [0.13 – 1.37] in absolute magnitude, or 11.3% [2.7 – 30.3] in relative magnitude (normalized to the intracardiac DF) with poor correlation (*R*^2^ = 0.11).

Further analysis of RI indices are included in Supplementary Figure 2, where we provide additional analyses comparing intracardiac and non-invasive DFs and RIs. Supplementary Figure 2A shows low correlation between intracardiac and non-invasive RI, which may reflect different causes for intracardiac recordings (far field, low contact) than non-invasive recordings (ventricular contamination, poor surface coverage). Nevertheless, Supplementary Figures 2A,B show that DF estimated non-invasively differed from invasive indices less in patients with higher RI values, and more in patients with lower RI. This was true for intracardiac (Supplementary Figure 2C) and non-invasive (Supplementary Figure 3D) RI measurements. This reinforces our selection of this marker to estimate the reliability of DF estimates.

### Systematic Comparison of Non-invasive and Basket DF, Filtered Using RI Threshold

We used RI calculated from spectral analysis, which measures stability of activation, as a filter to identify more stable signals. We used a RI threshold > 0.4 (intracardiac) and > 0.2 (non-invasive). RI thresholds were obtained by maximizing *R*^2^ analysis on our dataset after thresholding. RI thresholds were higher for intracardiac than non-invasive signals as the total spectral content was lower for ECGI signals after spectral filtering.

**FIGURE 3:**
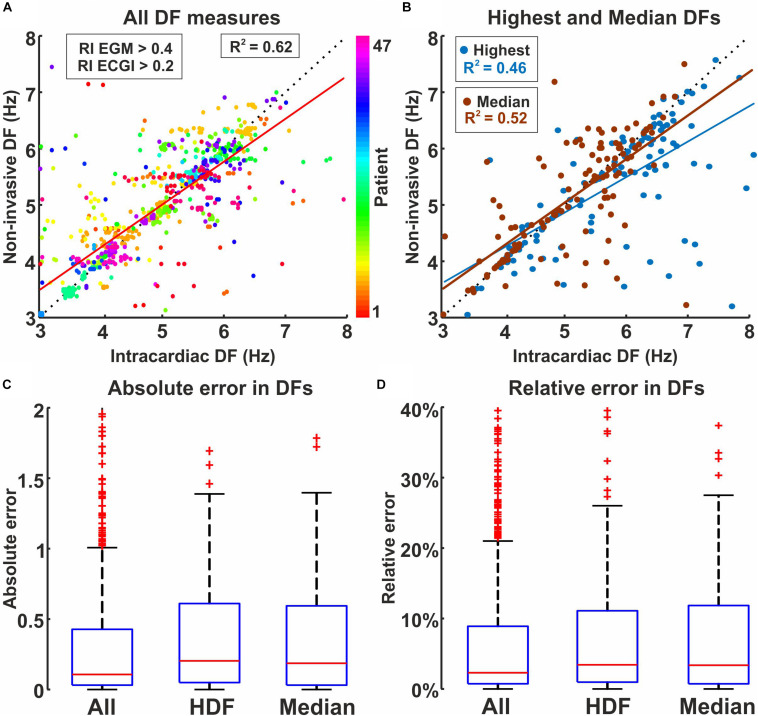
Intracardiac and non-invasive DF measures filtered by spectral organization (RI). **(A)** Dispersion plot by patient, using RI (intracardiac) > 0.4 and RI (non-invasive) > 0.2. **(B)** Dispersion plot coded by highest DF (blue) and Median DF (red). **(C)** Absolute and **(D)** relative (% of intracardiac measure) difference for all electrodes, Highest DF and Median DF.

Figure 3 shows intracardiac and non-invasive DF for pairs of tracings that fulfilled this RI threshold (967 signals from 123 episodes and 42 patients). Panel A depicts intracardiac vs. non-invasive DF comparison, color-coded by patient number. The absolute and relative deviation between non-invasive and basket DF measures was 0.10 Hz [0.03 – 0.42] and 2.3% [0.7–8.9], respectively (Panels C and D), with *R*^2^ = 0.62. These measures provided a Relative Difference Measurement Star (RDMS) of 0.075% [0.034 – 0.125]; and Relative Absolute Error (RAE) of 7.51% [3.77 – 14.4] across patients. We assessed inter-patient variability in the DF linear fit using repeated-measures one-way ANOVA to compare individual patients to the global linear regression fit. Notably, 19 out of 47 patients (40%) showed a significant difference (*p* < 0.05) between the patient-dependent and global trend fits.

Panel B shows median and highest DF (HDF) over both atria on a per-episode basis, calculated for 160 AF episodes (3 [2–5] per patient, 4.9 ± 1.4 s duration). Absolute and relative deviation between non-invasive and basket HDF measures were 0.20 [0.04 – 0.61] Hz and 3.5 [1.0 – 11.2]%, and the linear fitting *R*^2^ = 0.46, whereas the deviation for median DF was 0.19 [0.03 – 0.59] Hz and 3.4 [0.7 – 11.8]% (Panels C and D) and the linear fitting was *R*^2^ = 0.52. Highest DF and median DF were detected by ECGI with a difference from intracardiac electrodes of < 1.5 Hz except for 12/6 cases, respectively (1.2%/0.6%). Cases with deviations > 2.5 Hz had a poor number (< 10) of intracardiac/non-invasive signals to compare after RI threshold (Supplementary Figures 3, 4).

We also measured the accuracy of non-invasive maps to identify high DF regions revealed by intracardiac recordings, using WUI and WOI. High DF regions detected by ECGI produced a median false positive area of 27.6% [0.0 – 77.8] (WOI metric), whereas non-HDF regions detected by ECGI produced a median false positive area of 0.0% [0.0 – 50.0] of intracardiac high DF area (WUI metric), compared with intracardiac high DF regions.

### Ablation Outcome, Global and Regional Atrial Rate

We reasoned that patients more amenable to therapy may have more organized AF, and hence lower median DF. We further reasoned that within the lower overall DF milieu in such patients, sites of potential interest for targeted ablation may be the fastest, i.e., with highest DF.

**FIGURE 4:**
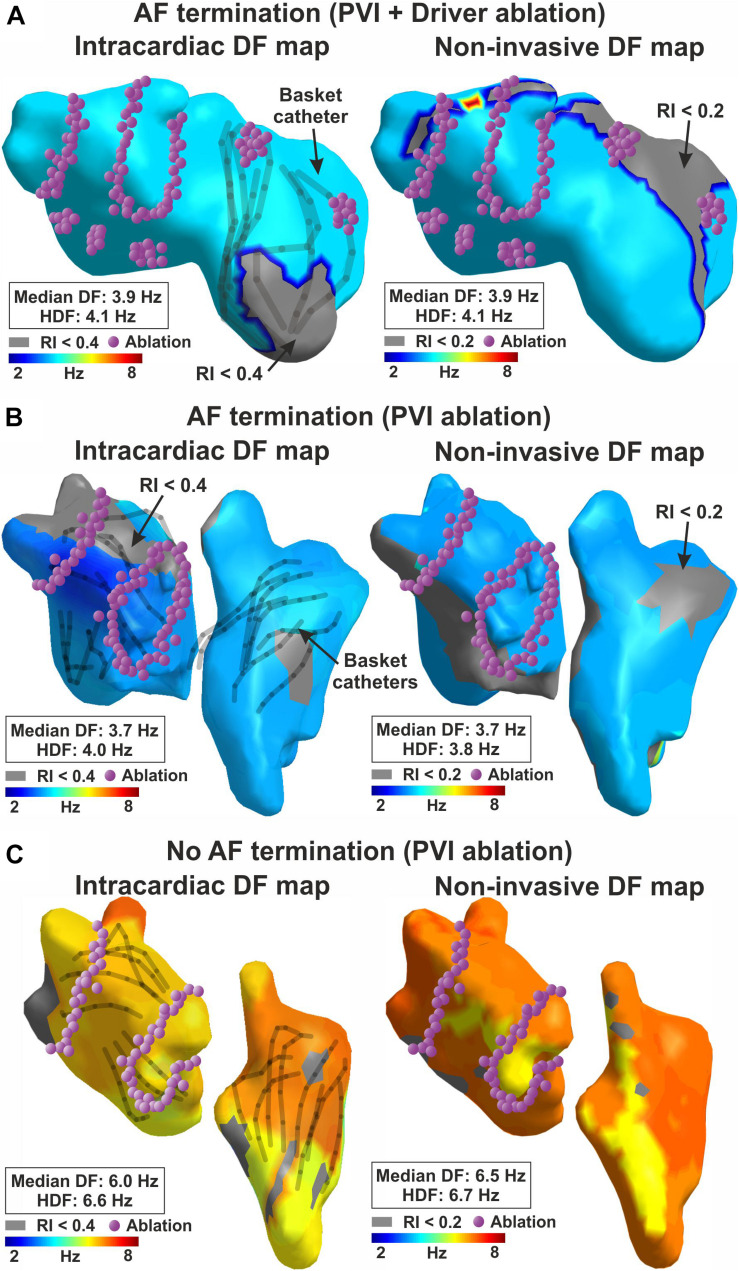
Dominant frequency maps and acute AF termination. Intracardiac (left) and non-invasive (right) DF map for **(A)** a patient with acute AF termination during guided ablation; **(B)** a patient with acute AF termination during PVI ablation; **(C)** no acute AF termination during PVI ablation.

Figure 4 shows simultaneous intracardiac and ECGI DF maps for two patients in whom AF did terminated acutely during ablation (Figures 4A,B) or did not terminate (Figure 4C). Intracardiac and non-invasive DF measures not fulfilling the RI threshold are depicted as gray. The first patient with acute termination during PVI + guided ablation showed slow rates across the atria: 3.9 Hz and 4.1 Hz of median DF and HDF in intracardiac maps, and 3.9 Hz and 4.1 Hz in ECGI maps, respectively. The second patient with acute termination during PVI only ablation showed also slow rates across the atria: 3.7 Hz and 4.0 Hz of median DF and HDF in intracardiac maps, and 3.7 Hz and 3.8 Hz in ECGI maps, respectively. Conversely, the third patient without AF termination during PVI only ablation had higher global activation rate: 6.0 Hz and 6.6 Hz for median DF and HDF, respectively, slightly faster in RA (∼6.5 Hz) than LA (∼6 Hz) as also shown on ECGI maps 6.5 Hz and 6.7 Hz, respectively). PVI ablation did not terminate AF in this patient.

**FIGURE 5:**
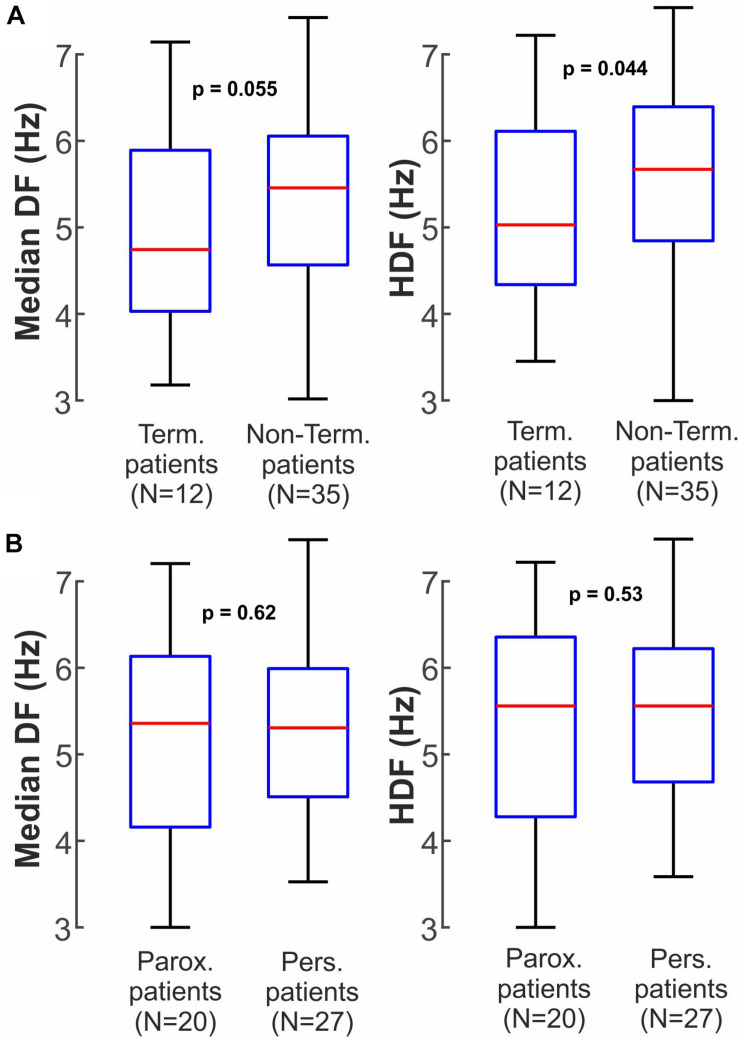
Non-invasive clinical Dominant Frequency measures between different patient groups. **(A)** Median DF and HDF for patients in which ablation did or did not terminate AF. **(B)** Median DF and HDF for paroxysmal and persistent AF patients.

Overall, Figure 5 shows this trend for higher ECGI DF in patients in whom ablation did not acutely terminate AF compared to those with AF termination. The resolution of our maps did not identify high-DF spots at sites where ablation acutely terminated AF on intracardiac nor ECGI DF maps. It is not clear if this represents small numbers, an artifact of DF analysis, map resolution that missed a small site of high DF, or the actual physiology. Patients with acute AF termination thus showed slightly lower overall activation rates reconstructed non-invasively than patients with no acute termination, in median DF (5.0 ± 1.1 Hz vs. 5.3 ± 1.0 Hz, *p* = 0.055) and HDF (5.2 ± 1.1 Hz vs 5.6 ± 1.0, *p* = 0.044). No significant differences were found using this approach for classical AF classification such as paroxysmal and persistent patients (Figure 5B), neither in median DF (5.2 ± 1.2 Hz vs 5.2 ± 0.9 Hz, *p* = 0.62) nor in HDF (5.4 ± 1.2 Hz vs 5.5 ± 0.9 Hz, *p* = 0.53).

Non-invasive DF measures were also calculated between different patient groups in Supplemental Figures 5, 6. Differences between terminating and non-terminating patients where only present in persistent patients (median DF: 4.9 ± 0.9 Hz vs 5.4 ± 0.8 Hz, *p* = 0.005; highest DF: 5.1 ± 0.9 Hz vs 5.7 ± 0.9 Hz, *p* = 0.002). Paroxysmal patients did not show differences in median DF (5.1 ± 1.4 Hz vs 5.2 ± 1.1 Hz, *p* = 0.98) or Highest DF (5.4 ± 1.3 Hz vs 5.4 ± 1.1 Hz, *p* = 0.90).

Patients in whom AF terminated by ablation showed lower DF in non-invasive maps than patients in whom AF did not terminate in both AF ablation protocols (Supplementary Figure 6). In patients receiving PVI + substrate ablation, median DF were 5.1 ± 1.1 Hz vs 5.5 ± 0.8 Hz, *p* = 0.035, while highest DF were 5.3 ± 1.0 Hz vs 5.7 ± 0.8 Hz, *p* = 0.034. Patients receiving PVI-only ablation showed a similar (but non-significant) trend for median DF of 4.7 ± 1.0 Hz vs 5.1 ± 1.0 Hz, *p* = 0.18 and for highest DF of 4.9 ± 1.1 Hz vs 5.4 ± 1.1 Hz, *p* = 0.15).

**FIGURE 6:**
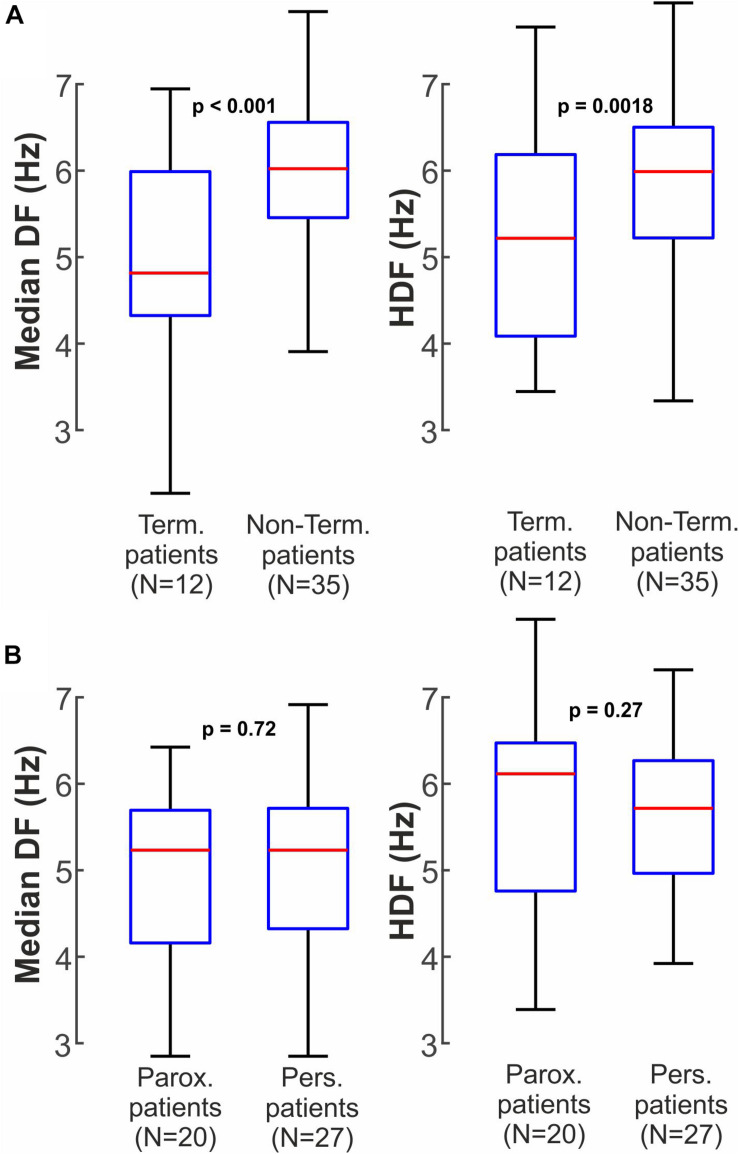
Intracardiac clinical Dominant Frequency measures between different patient groups. **(A)** Median DF and HDF for patients in which ablation did or did not terminate AF. **(B)** Median DF and HDF for paroxysmal and persistent AF patients.

Finally, we examined DF between patients with and without AF termination from intracardiac data (Figure 6). In intracardiac recordings, patients with AF termination by ablation showed lower overall DF than patients without termination, for both median DF (4.5 ± 0.9 Hz vs 5.3 ± 0.8 Hz, *p* < 0.001) and highest DF (5.3 ± 1.1 Hz vs 5.8 ± 0.9 Hz, *p* = 0.0018). Similarly to non-invasive measures from Figure 5B, no significant differences were found in intracardiac DFs between paroxysmal and persistent patients (Figure 6B) in median DF (5.0 ± 0.9 Hz paroxysmal vs 5.0 ± 0.9 Hz persistent, *p* = 0.72) nor in highest DF (5.8 ± 1.2 Hz paroxysmal vs 5.6 ± 0.9 Hz persistent, *p* = 0.27).

## Discussion

In this study, simultaneous intracardiac and non-invasive measures of atrial activation rate in AF were compared in a cohort representing diverse clinical types of AF. We found agreement between intracardiac and non-invasive measures of dominant frequency during AF, that was dramatically improved by secondary markers of stability such as regularity index. We identified spectral signatures, or ‘phenotypes’ for patients who did and did not achieve the clinical outcome of the acute AF termination by ablation. Future work should extend this to endpoints such as long-term freedom from arrhythmias, and compare these results using different filtering strategies.

### Dominant Frequency and Atrial Fibrillation Management

Optical mapping of explanted hearts shows that atrial activation rate may play a fundamental role in maintaining AF (Kalifa et al., 2006; Filgueiras-Rama et al., 2012). In many studies, regions of higher activation rate appeared to drive fibrillatory activity in the rest of atrial tissue, and in some studies their ablation terminated AF (Sanders et al., 2005; Atienza et al., 2014). Of note, this was found in patients with paroxysmal AF but not those with persistent AF.

Electrical remodeling favoring progression of AF from paroxysmal to persistent then permanent may be linked with an increase in atrial activation rate (Martins et al., 2014). Therefore, patients presenting with lower DF measurements on the ECG have been linked with better long-term outcome of ablation procedures (Murase et al., 2020). Therapy based on the identification and ablation of fastest activated regions based on intracardiac estimation of DFs have been shown to be equivalent to pulmonary vein ablation (Atienza et al., 2014) particularly in relatively early paroxysmal AF.

Comparing the findings of this study to the prior literature, therefore, one could conclude that both lower global DF measurements and the existence of DF gradients within the atria can simultaneously predict ablation success. Further studies are needed to investigate whether previously reported DF gradients may be exaggerated by the use of adenosine, which is known to highlight differences in atrial activation rate (Atienza et al., 2006), and thus may not be expected in this study.

Early stages of AF exhibit lower activation frequencies and less complex propagation patterns. Notably, DF may better discriminate acute outcome in patients with persistent AF than paroxysmal AF. This supports differential electrical substrate remodeling detectable by DF, in which earlier AF (paroxysmal) show lower activation frequencies and less complex propagation than later stage AF. Lower activation frequency (longer cycle length) corresponds to larger wavelength and therefore a smaller number of co-existing wave-fronts (Kneller et al., 2005). These findings agree with other studies that lower complexity measured as number of different AF reentrant drivers correlates with better ablation outcomes (Haissaguerre et al., 2014; Rodrigo et al., 2020).

### Non-invasive Identification of AF Activation Rate

Electrocardiographic imaging has been used to non-invasively map electrical activity of the whole epicardium. Haissaguerre et al. (2014) used this technique in 103 persistent AF patients to guide the ablation procedure, showing acute termination rate of 75% for persistent and 15% for long-standing AF patients, and showing that the number of targeted regions was related to the degree of AF progression, and inversely related to the probability of AF termination. These results agree with our findings.

Electrocardiographic imaging may be more robust in classifying AF patients than in quantifying and mapping specific propagation patterns in AF. Recent studies raise methodologic questions on the technical ability of ECGI to reconstruct cardiac electrical activity. Duchateau et al. showed that local activation time diverges between non-invasive and catheter-based epicardial recording (Duchateau et al., 2018). Methodologic factors such as bipolar vs. unipolar recordings, or biophysical limitations of the inverse resolution may prevent signals reconstructed by ECGI from having temporal and/or spatial precision that accurately represents local activations to reproduce isochronal maps (Rodrigo et al., 2017b; Rudy, 2019). Our findings show that non-invasively acquired frequency organization may be used to improve the accuracy of non-invasive cardiac mapping of AF. DF analysis in AF is intrinsically more stable than activation times, since it does not depend on a single fiducial point (activation time) to define a series of isochrones for activation or phase mapping. Frequency mapping summarizes a series of temporal voltage distributions in a single DF value, and thus should be more robust to biophysical limitations of the inverse problem than activation time. This was reported in previous studies in which we found that displacements and rotations greatly reduced agreement of inverse-solution computed signals with original signals, yet preserved frequency information as the concordance of the HDF region remained almost stable (Rodrigo et al., 2017a).

We found that spectral organization indicated by the Regularity Index (RI) is a useful index of the reliability of a specific estimated frequency. Lower RI implies lower energy of that DF peak, and filtering out such peaks improved the accuracy of non-invasive estimates. Low RI may result from factors that indicate non-local activity, including far field contributions, ventricular artifacts, low electrode contact or electrical noise. Local mechanisms such as wave fractionation or competing AF mechanisms may also produce low RI. Previous findings of our group and others suggest that intracardiac DF measurements can be affected by far-field contributions particularly when the basket electrodes are at a distance from the atrial wall (Martinez-Mateu et al., 2019, 2018). In the present study, we used bipolar recordings in order to diminish such far-field artifacts.

### Clinical Perspective of Non-invasive AF Mapping

To date, non-invasive AF mapping through ECGI has been used to guide and plan AF ablation, specifically by identifying those atrial regions harboring reentrant drivers (Haissaguerre et al., 2014). This study broadens the application of non-invasive mapping to identify AF ‘phenotypes’ which may be relevant to acute and long-term success from therapy. Our results suggest that global measures such as median DF may also be valuable markers of AF outcome, and future studies should evaluate whether this can be obtained from simpler approaches such as Body Surface Potential Maps or even the classical ECG. The current high resolution ECGI approach may be ultimately needed to classify long-term outcomes, a focus of ongoing studies, and to improve clinical phenotypes such as paroxysmal and persistent AF. Non-invasive activation rate mapping of AF may also help to tailor therapies including drugs to electrical phenotypes detected in specific patients. Clearly, prospective clinical trials are needed to test each of these hypotheses.

### Limitations

Results from this paper were obtained with patients from 2 different institutions and under different AF therapies (PVI and guided ablation) and demographic differences. Nevertheless, results obtained for non-invasive DF mapping were consistent between and within each cohort, showing the robustness of non-invasive atrial activation rate mapping in different types of AF patients: paroxysmal, persistent, long-standing and valvular AF. On the other hand, the specific thresholds we used for RI filtering may differ in other cohorts. Finally, long-term clinical results of ablation in these patients vary with different ablation protocols. We selected AF termination as it is a common acute endpoint for ablation, and because it is independent on differences in post-ablation medications, long-term lesion recovery, drug adherence and AF progression.

## Conclusion

Non-invasive characterization of atrial rate in AF, filtered by spectral measures of organization, identifies regional activation rates on intracardiac mapping. Non-invasive analysis could provide a mechanistic basis for clinical phenotypes such as patients who are likely to experience AF termination by ablation, or ultimately those who may experience long-term arrhythmia freedom from ablation or drug therapy.

## Data Availability Statement

The raw data supporting the conclusions of this article will be made available by the authors, without undue reservation.

## Ethics Statement

The studies involving human participants were reviewed and approved by Stanford Hospital, Hospital General Universitario Gregorio Marañon. The patients/participants provided their written informed consent to participate in this study.

## Author Contributions

Only those who have made an important contribution to the study and are thoroughly familiar with the primary data are included as authors, and all authors are responsible for the contents and have read and approved the manuscript and conform to the Uniform Requirements for Manuscripts Submitted to Biomedical Journals published in Annals of Internal Medicine 1997; 126:36–47.

## Conflict of Interest

MR, IH-R, and AL: equity from Corify Care SL. SN: compensation for services from up to date, Abbott Laboratories, American College of Cardiology Foundation. Intellectual property rights from University of California Regents and Stanford University. Grant support from the National Institutes of Health (R01 HL83359, R01 HL149134, and K24 HL103800). The remaining authors declare that the research was conducted in the absence of any commercial or financial relationships that could be construed as a potential conflict of interest.
