# Supplementary material for: Non-invasive Spatial Mapping of Frequencies in Atrial Fibrillation: Correlation With Contact Mapping
Source: Front Physiol. 2021 Jan 6;11:611266. doi: 10.3389/fphys.2020.611266 (PMC7873897; doi:10.3389/fphys.2020.611266)
Supplement: Supplementary file 1 [file Data_Sheet_1.PDF]

Supplementary Material

1 Supplementary Figures

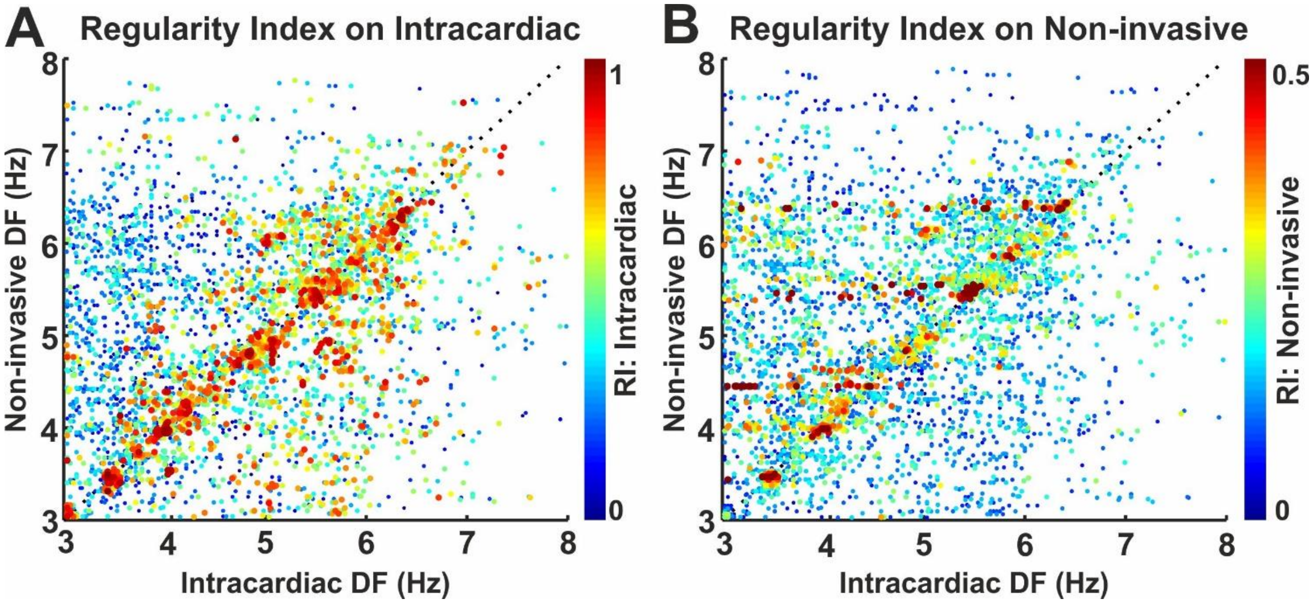

Supplementary Figure 1. Comparison of intracardiac and non-invasive DF measures. Dispersion plot between the intracardiac and non-invasive DF measures, for all the signals registered (N=4566), color-coded based on Intracardiac **(A)** and Non-invasive **(B)** Spectral Organization (Regularity Index, RI).

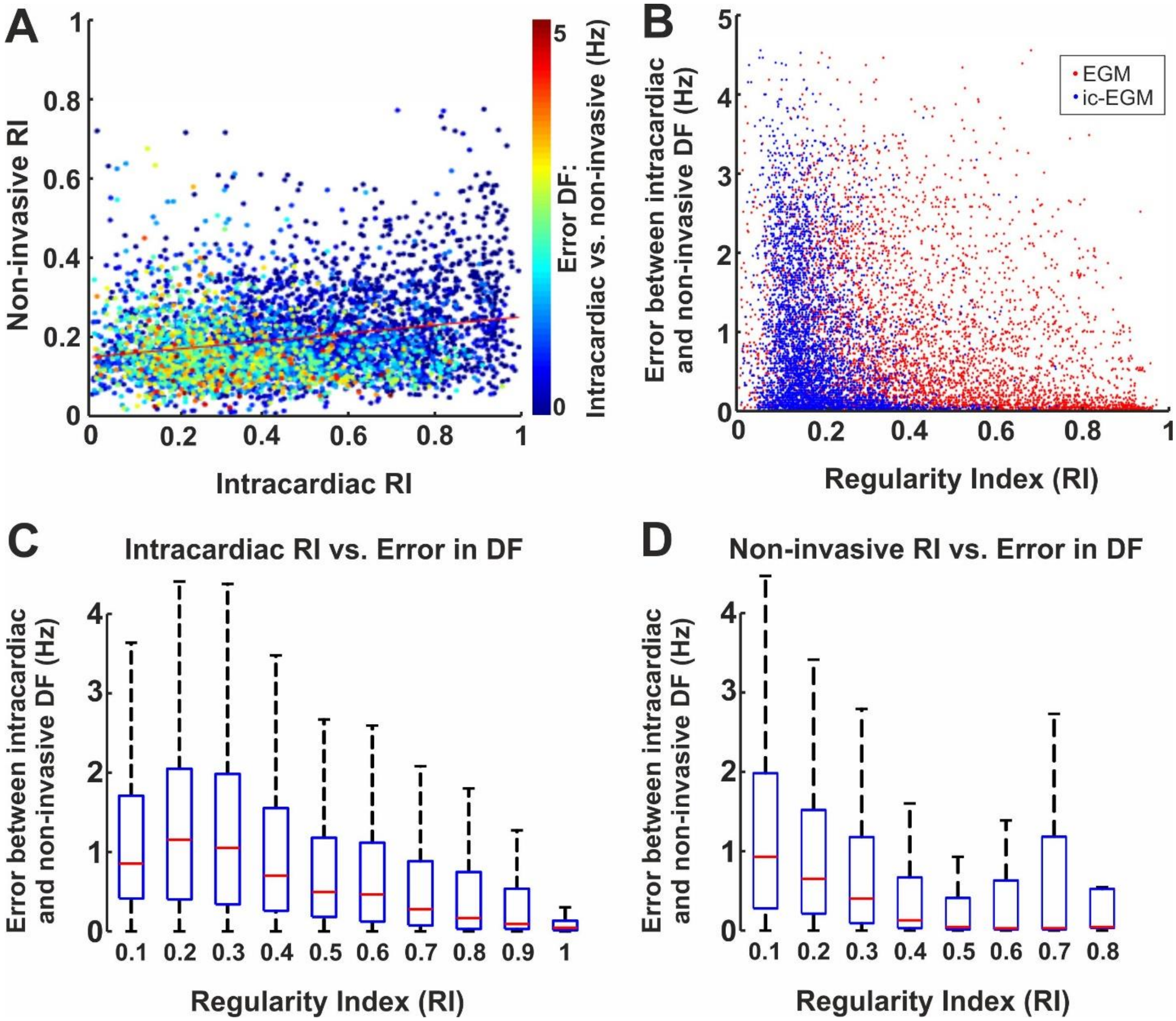

Supplementary Figure 2. Comparison of intracardiac and non-invasive RI measures. A. Dispersion plot between the intracardiac and non-invasive RI measures, for all the signals registered (N=4566), color-coded based on difference between intracardiac and non-invasive DF measure. **B**. Dispersion plot between the intracardiac (red) and non-invasive RI measures (blue) vs. the difference between intracardiac and non-invasive DF measure, for all the signals registered (N=4566). **C**. Difference between intracardiac and non-invasive DF measure in batched intracardiac RI indexes. **D**. Difference between intracardiac and non-invasive DF measure for batched intracardiac RI indexes.

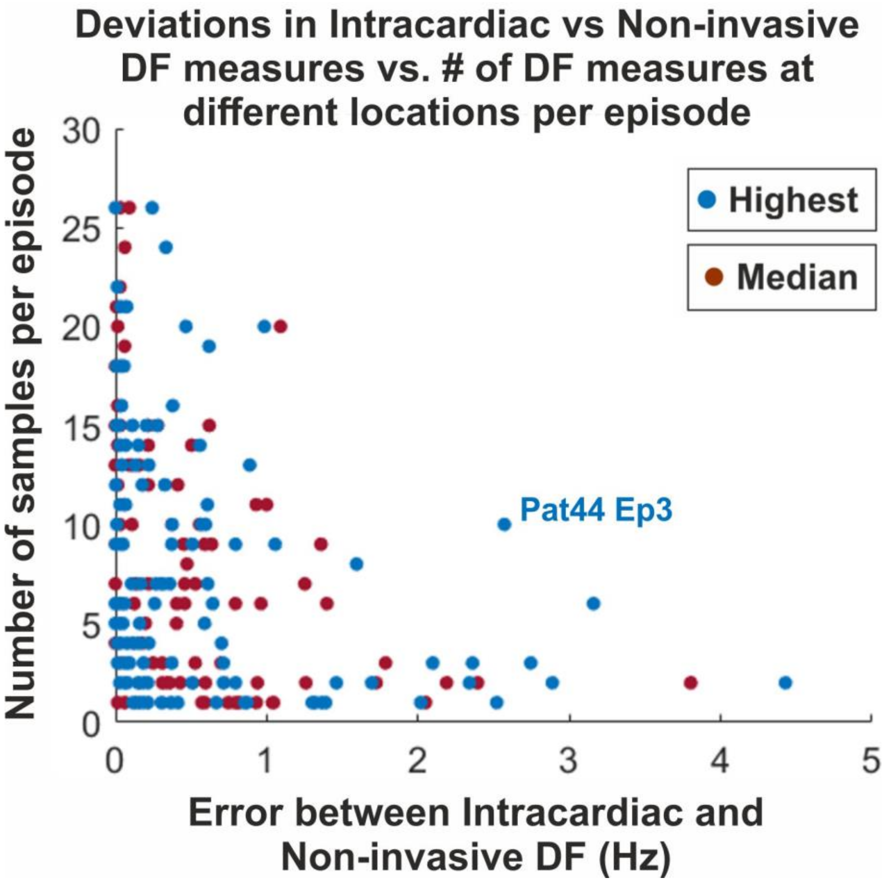

Supplementary Figure 3. Deviation in Intracardiac vs Non-invasive DF measures and the number of DF measures at different.

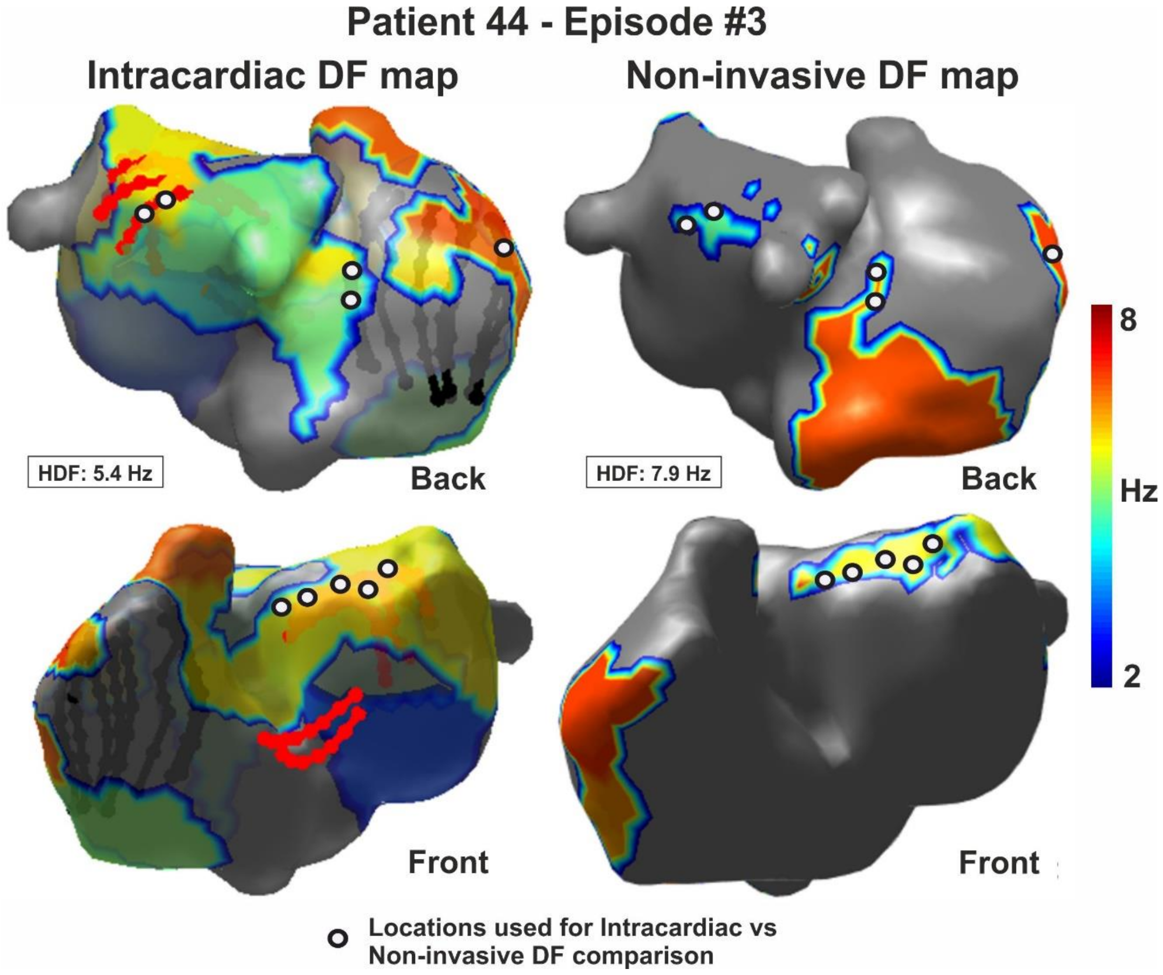

Supplementary Figure 4. Intracardiac (left) and Non-invasive (right) DF map for the episode #3 of patient 44. Only 10 locations were available for comparison between Intracardiac and Non-invasive DF.

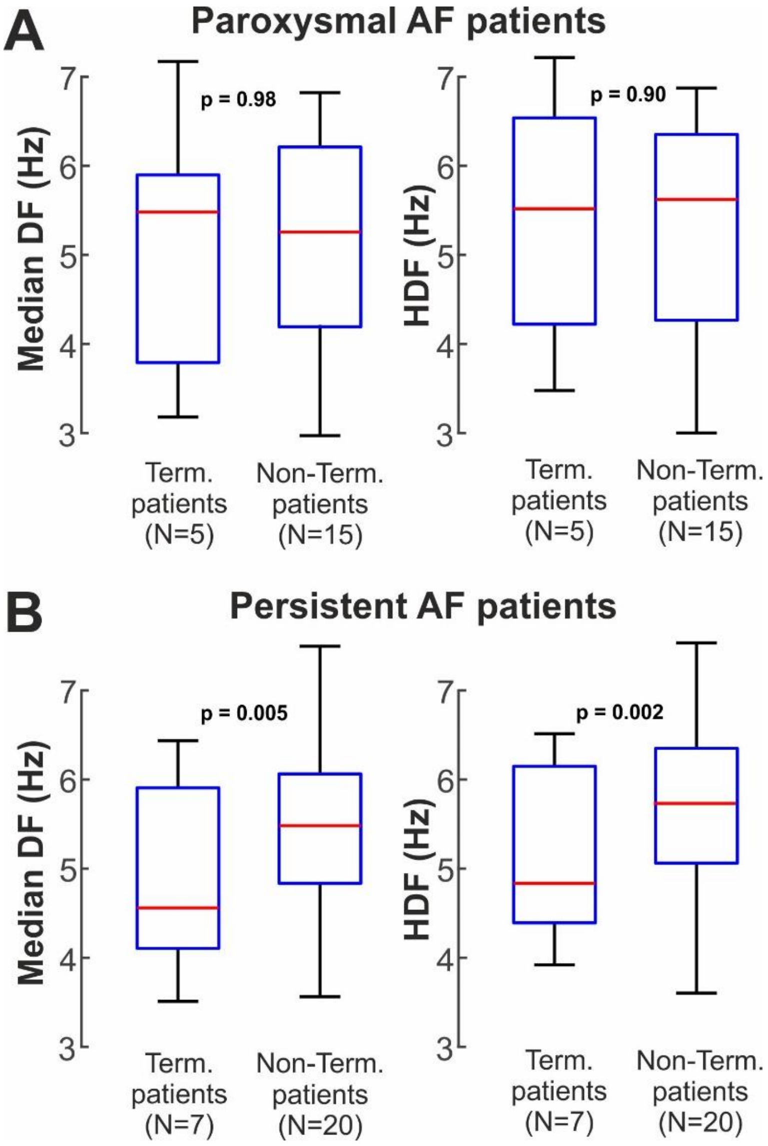

Supplementary Figure 5. Non-invasive clinical Dominant Frequency measures depending on AF classification. A. Median DF and HDF for paroxysmal AF patients in which ablation did or did not terminated AF. **B.** Median DF and HDF for persistent AF patients in which ablation did or did not terminated AF.

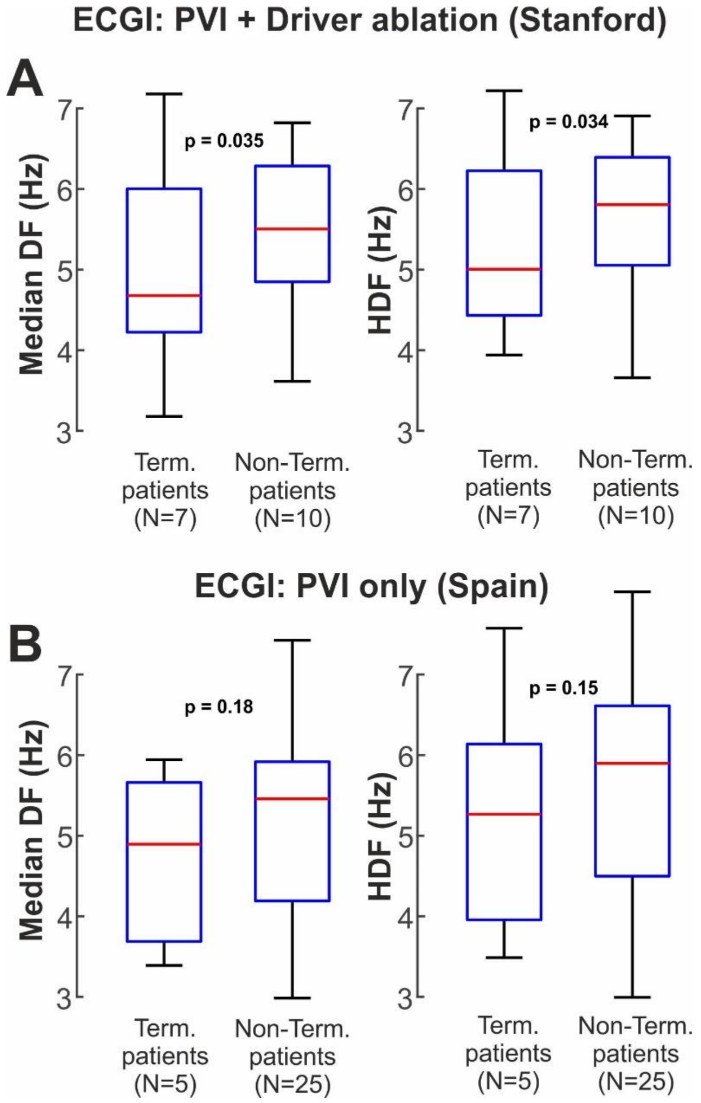

Supplementary Figure 6. Non-invasive clinical Dominant Frequency measures depending on ablation strategy. Median DF and HDF for patients in which PVI + driver ablation **(A)** or PVI only ablation **(B)** did or did not terminated AF.
